# Supplementary material for: LIonomers-New Generation of Ionomer: Understanding of Their Interaction and Structuration as a Function of the Tunability of Cation and Anion
Source: Polymers (Basel). 2023 Jan 10;15(2):370. doi: 10.3390/polym15020370 (PMC9865942; doi:10.3390/polym15020370)
Supplement: Supplementary file 1 [file polymers-15-00370-s001.zip › polymers-2056881-supplementary.pdf]

Supplementary Materials

1. Elemental Analysis Spectra (EDX-TEM)

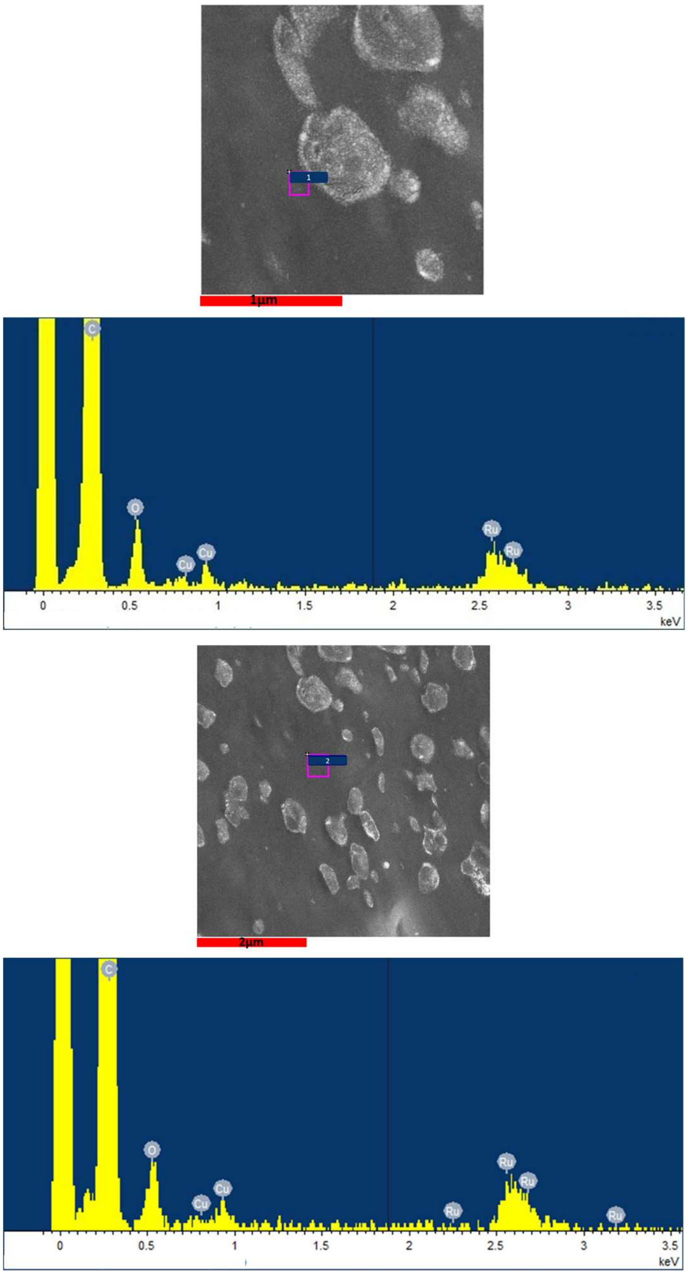

Polymer matrix within L-P^{+}DEP-10

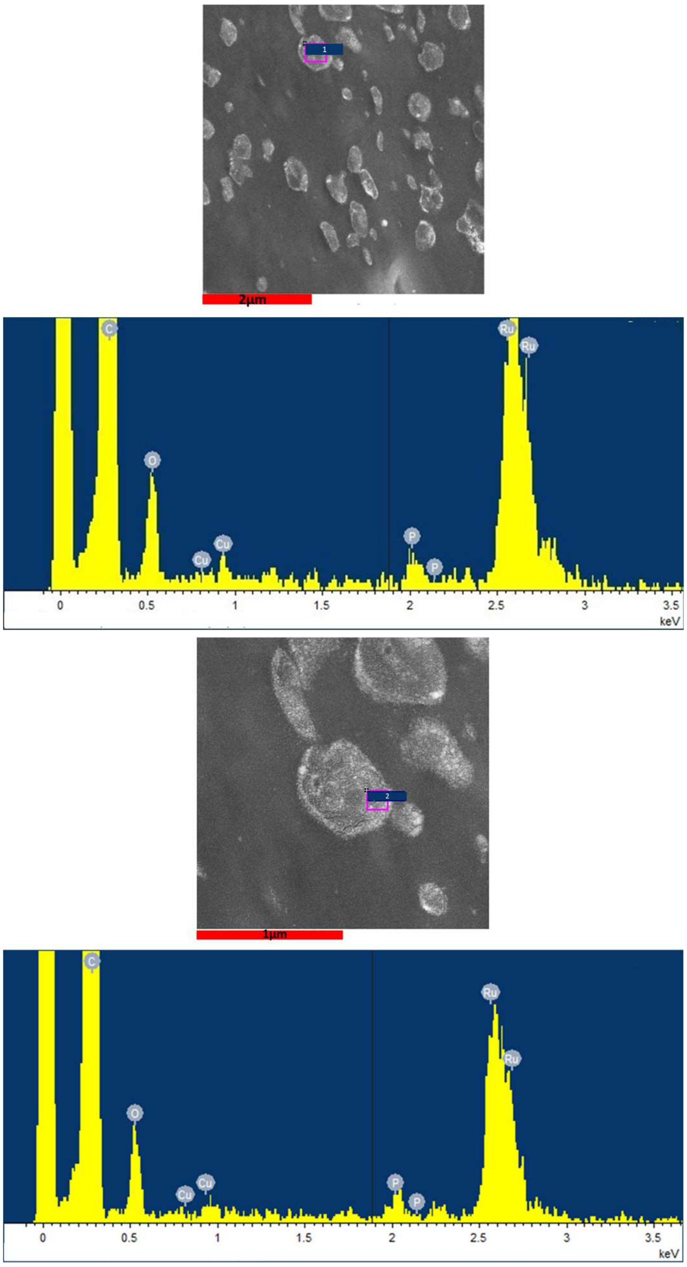
1
2µm
C
O
Cu
Cu
P
P
Ru
Ru
0
0.5
1
1.5
2
2.5
3
3.5
keV
2
1µm
C
O
Cu
Cu
P
P
Ru
Ru
0
0.5
1
1.5
2
2.5
3
3.5
keV

L-P$^+$DEP-10

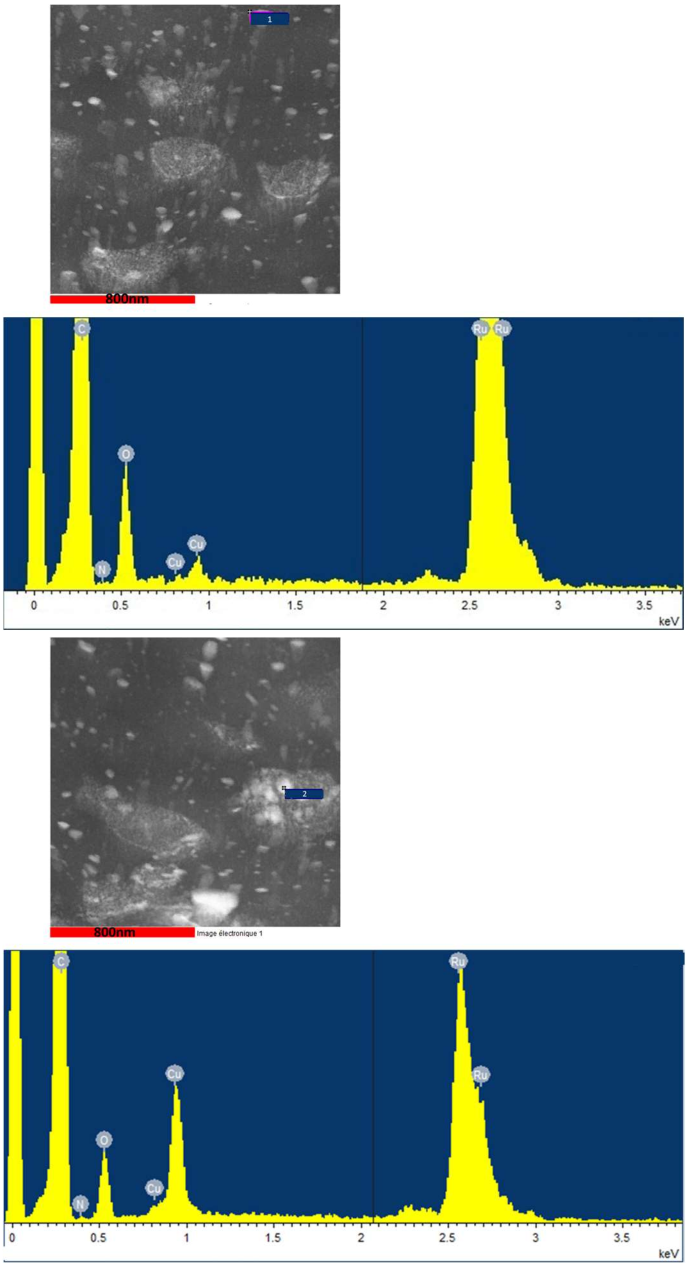
1
800nm
C
O
N
Cu
Ru
0
0.5
1
1.5
2
2.5
3
3.5
keV
2
800nm
Image électronique 1

L-EMIM Ac-10

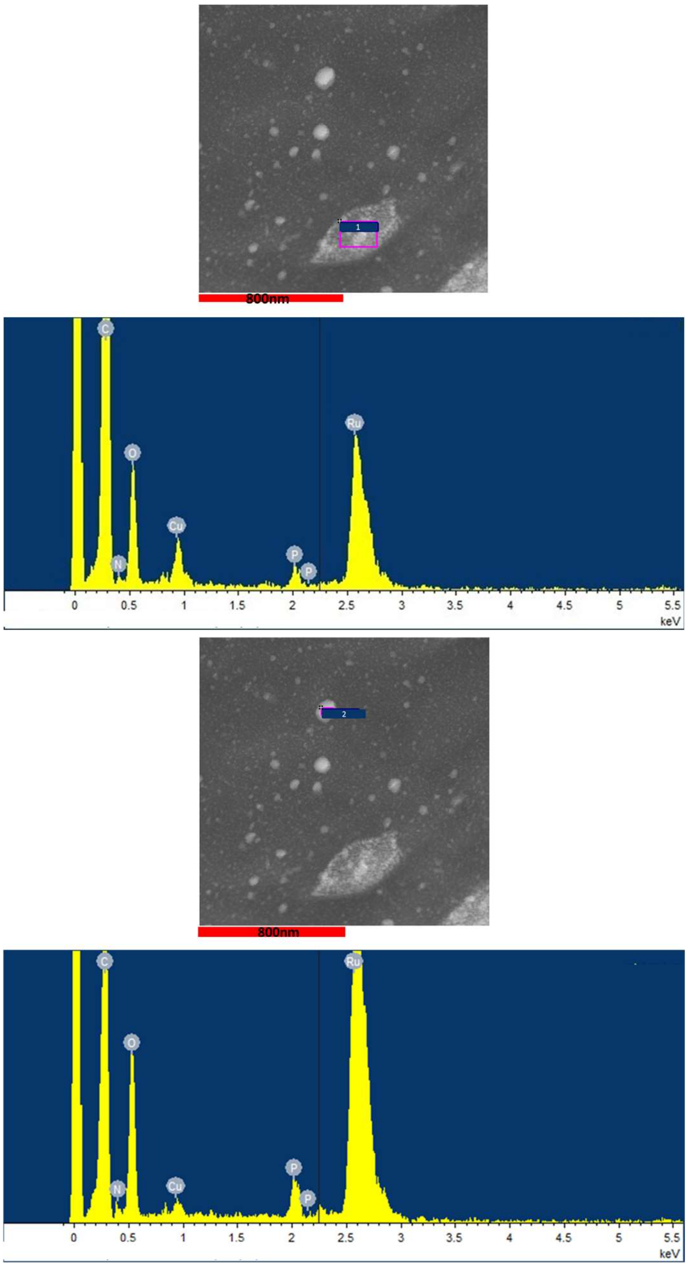

L-EMIM DEP-10

Figure S1. Elemental analysis spectra (EDX) of LIonomers performed on fractured surfaces observed by TEM.

2. DSC thermograms of PPgMA and related LIonomers

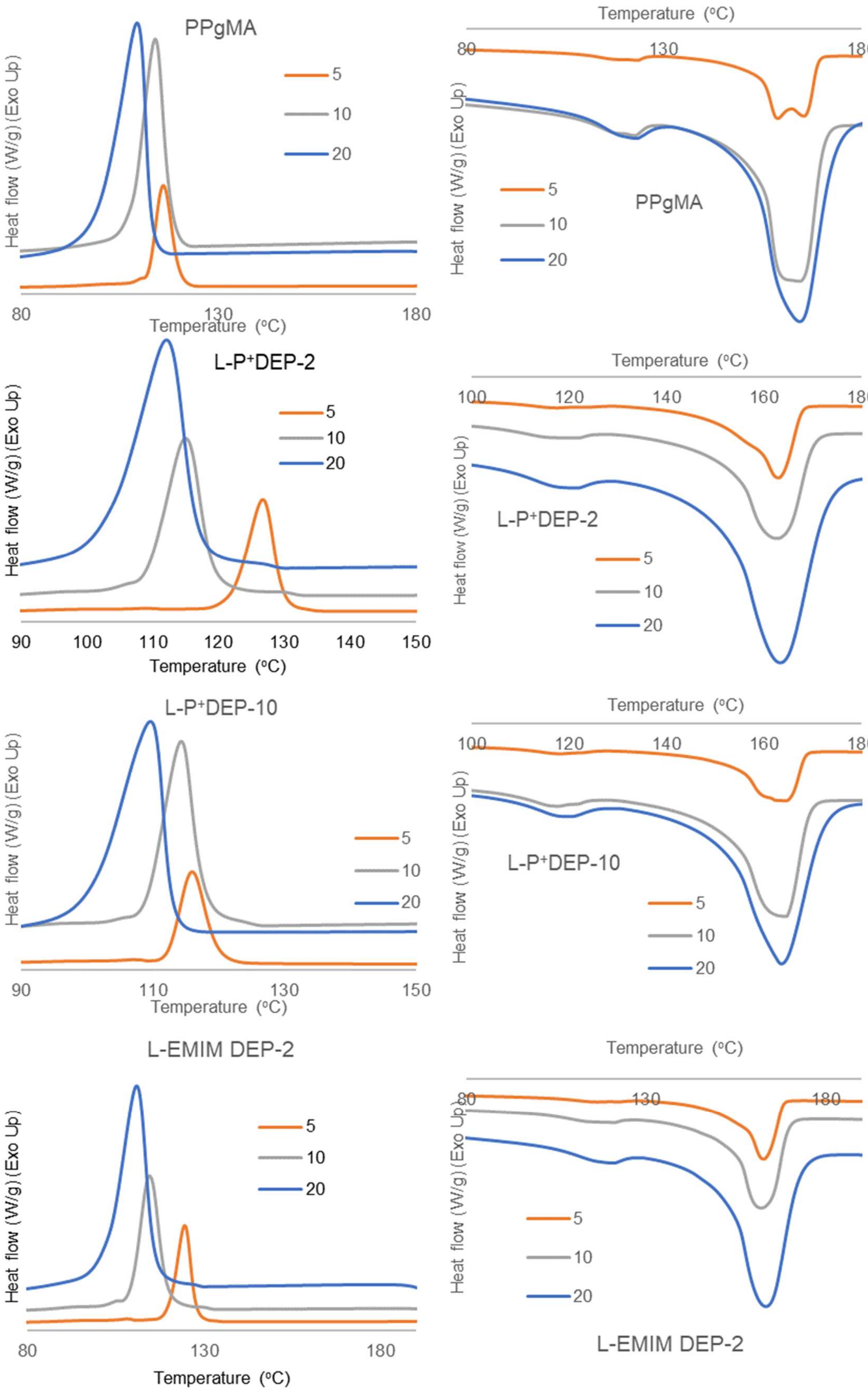

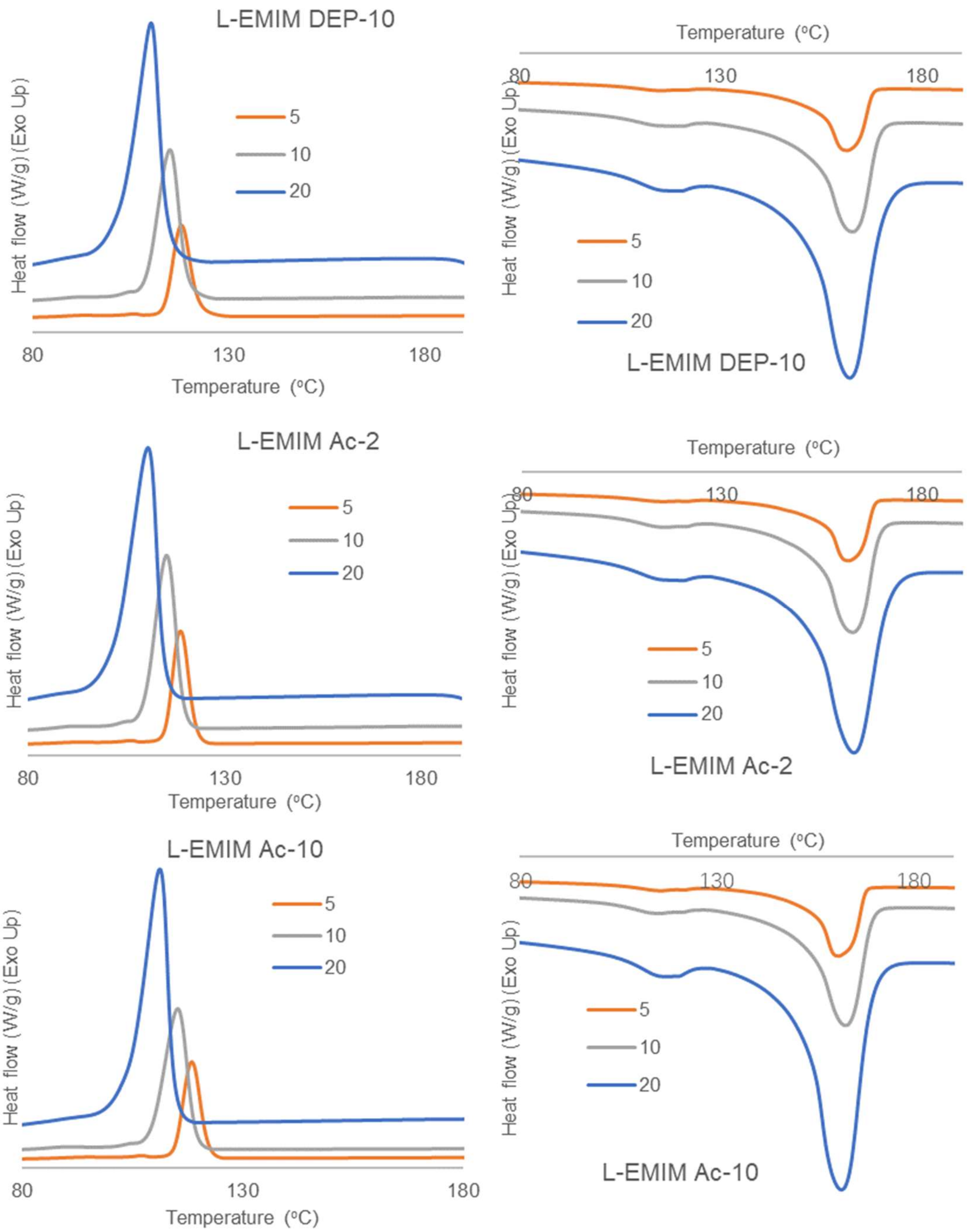

Figure S2. DSC traces of PPgMA and related LIonomers prepared with various ionic liquids (2 and 10 wt.%). Melting and crystallization phenomenon was observed for different heating and cooling rates (5, 10, and 20 K·min^{-1}) under nitrogen atmosphere.

3. ln [-ln(1-X_t)] vs. ln t plots according to Jeziorny's non-isothermal crystallization model

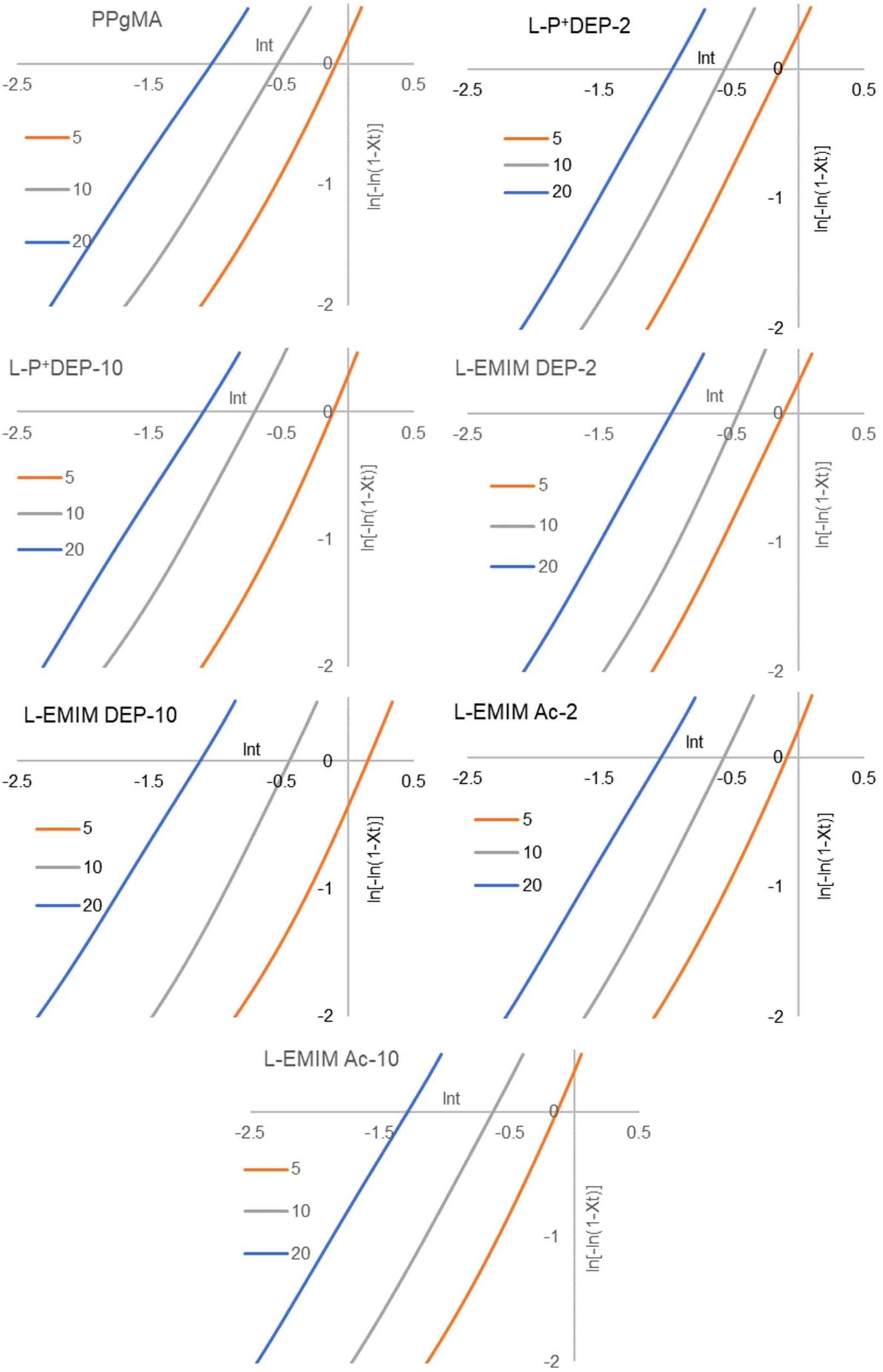

Figure S3. $\ln[-\ln(1-X_t)]$ vs. ln t plots according to Jeziorny's crystallization model for neat PPgMA and related LIonomers based on various ionic liquids (2 and 10 wt.%) for different cooling rates (5, 10, and 20 K·min^{-1})

4. Detailed parameters of non-isothermal crystallization process in accordance with Jeziorny's model

Table S1. Parameters of non-isothermal crystallization process based on Jeziorny's model.

Samples	Φ (K/min)	$t_{1/2}$(min)	n	Z_t	T_m(°C)	T_c(°C)	X_c(%)
	5	0.77	2.01	1.17	159.0/165.6	116.1	34.1
PPgMA	10	0.47	1.78	2.68	164.2	114.1	35.5
	20	0.28	1.65	5.55	164.5	109.5	31.6
	5	0.74	2.01	1.28	163.0	126.8	37.6
L-P^+DEP-2	10	0.48	1.88	2.81	162.8	115.0	34.7
	20	0.32	1.76	5.29	163.4	112.2	33.6
	5	0.76	2.06	1.22	164.5	116.0	35.1
L-P^+DEP-10	10	0.41	1.81	3.54	164.7	114.3	34.0
	20	0.27	1.65	6.16	163.7	109.6	32.1
	5	0.72	1.98	1.33	162.9	124.6	36.8
L-EMIM DEP-2	10	0.50	1.94	2.65	162.3	114.6	34.3
	20	0.31	1.81	5.66	163.4	111.0	33.2
	5	0.87	2.04	0.92	161.6	118.0	35.3
L-EMIM DEP-10	10	0.49	1.85	2.56	162.9	115.1	34.0
	20	0.28	1.69	5.98	162.2	110.2	33.4
	5	0.77	2.03	1.16	161.9	118.8	34.2
L-EMIM Ac-2	10	0.47	1.92	2.92	162.9	115.2	33.7
	20	0.29	1.71	5.90	163.2	110.5	34.6
	5	0.72	2.30	0.84	161.0	118.5	36.9
L-EMIM Ac-10	10	0.44	1.86	3.20	162.8	115.3	35.8
	20	0.22	1.73	9.35	161.7	111.2	35.0

Note: φ: cooling rate (K.min^{-1}), T_m: melting temperature (°C), T_c: crystallization temperature (°C) and X_c: crystallinity (%).
